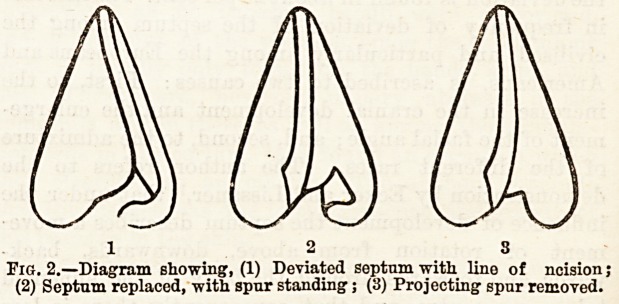# Progress in Surgery

**Published:** 1897-01-30

**Authors:** 


### PROGRESS IN SURGERY.

### DISEASES OF THE UPPER AIR PASSAGES.

(*Continued from p.* 268.)

**Deviations, Spurs, and Ridges of the Septum.**—In his contribution on the discussions on this subject, at the eighteenth American Laryngological Association, John O. Roe[8] states that he considers that some points have been overlooked which it is necessary to consider in order to explain why deviations of the septum are so frequent in their occurrence, and often so exceedingly peculiar as regards their form, extent, and location. Of the predisposing causes the two chief factors are diathesis and racial characteristics. As regards the question of diathesis, it is only necessary to point to the fact, verified by nearly all observers, that deflections of the nasal septum are exceedingly common among persons suffering from the strumous syphilitic, tubercular, or rachitic diathesis; and Harrison Allen[9] calls attention to the influence of cretinism in their production. In reference to racial characteristics, we note, for instance, among Europeans and Americans, the percentage varies from 60 to 80 per cent., while among the Mongolians, Polynesians, and the Africans the deviation is found in about 20 per cent. The increase in frequency of deviation of the septum among the civilised, and particularly among the Europeans and Americans, is ascribed to two causes: First, to the increase in the cranial development and the enlargement of the facial angle; and, second, to the admixture of the different races. The author refers to the demonstration by Ecker and Lissauer,[10] that under the influence of development the septum describes a movement of rotation from above, downwards, backwards, and forwards around the body of the sphenoid taken on a centre, and that, consequently, there is less room for the septum anteriorly.

Further studies in this direction show that an increase in the frequency of deviations of the septum corresponds very closely with the degree of admixture of the different races. This admixture in the more civilised countries, occurring largely as the result of conquest and of immigration, has been taking place from the earliest times, until modern Europe and America of to-day represent this admixture of the races in the highest degree.

He cites Delavan,[11] who emphasises the greater prevalence of deviated septa among the civilised than among savage races, and also points out the fact that the aquiline type of nose, as illustrated in the Slav, the Hebrew, and the ancient Roman, is particularly apt to be associated with deflections. A notable exception to this observation is the fact that the North American Indian, while possessing an aquiline nose, rarely has deflection of the septum. This is to be explained by the fact that the Indian belongs to a primitive and pure race, and also by their higher physical development on account of their outdoor life; and, as has been pointed out by Catlin,[12] by the habit among the Indian mothers of closing the mouths of their children, and compelling them to breathe entirely through their noses from their earliest infancy, which conduces to the proper development of the nose.

As exciting causes, Roe gives (*a*) defective development, (*b*) diseases of the septum, and (*c*) diseases of other portions of the nose.

Roe arrived at the following conclusions:—

1. That deviations of the septum are produced by a variety of causes operating upon different persons; and that upon the same person several different influences may be operating at the same time.

2. That heredity plays a very important part as a predisposing cause, not only by the dyscrasias which may be transmitted, but by the blending of different races in the composite type, which brings about an infinite variation in the conformation of the osseous and cartilaginous structures.

3. That the three local causes most frequently producing deviation, spurs, and ridges of the septum are trauma, nasal obstruction, and unequal growth of the different component parts of the vomer. The last-mentioned cause is itself produced mainly by local malnutrition or diseased conditions of the structures of the nasal passages, inducing an unequal development of the two sides of the septum, which causes a bulging or bending to the side of greatest development. The fact that the vomer is composed of two laminæ, separated by a plate of fibro-cartilage, which is continued forward to form the triangular cartilage of the nose, readily explains how this unequal development takes place.

**Treatment.**—The diversity of opinion as to the gravity of septal deviations and spurs, and the best method of dealing with them, indicates that no method can be at present accepted as altogether satisfactory. True, if the successes reported by the advocates of each method can be accepted in their entirety, we should have no great difficulty in dealing with the condition, inasmuch as we should have many excellent methods from which any one might be chosen. A. W. Watson[13] reminds us that the symptoms caused by a deviation of the septum are not confined to those arising from obstructed respiration. They include interference with drainage, obstruction of the outlets of the accessory sinuses, symptoms due to the abnormal size of the opposite nasal chamber, and that large class of affections caused by contact or pressure known as reflex. The relief of respiration, therefore, is not all that is called for in these cases. In his address to the American Laryngological Association, the author refers to the most important operations that aim at straightening the septum, viz.: Adams's, forcible breaking of the septum, followed by metal plugs or splints; Steele's, incisions by the stellate punch, followed by plugs; Roberts' multiple incisions by the knife, the fragments retained by pins; Ingals's, excision of a wedge-shaped piece and suture; and Roe's, crushing by special forceps, followed by antiseptic plugs. Allen's operation, in which the base of the septum is detached beneath the mucous membrane and the septum moved toward the open side and retained by a plug, does not aim at reducing the deviation, but simply equalising its encroachment on the nasal chambers. By all these methods, with the exception of Ingals's and Allen's, the curved septum is forced into a straight line, for which it is obviously too large, and for that reason the results must be but temporary in the majority of cases, where the deviation is at all marked.

The requirements of an operation for the correction of a deviated septum, in order that the result may be permanent, are considered by Watson in two parts: The operation proper, or the method of reducing the deviation, and the means for holding the parts in position until healing has taken place.

Many of the operations devised have been unsuccessful because they lose sight of the fact that a deviated septum is larger than a straight one, and make no provision for reducing the amount of tissue. The first step, therefore, is to reduce the septum to a size that will fit into a straight line between the points of attachment. This can be accomplished by excising a portion of tissue in the general line of deviation. If the deviation is horizontal, an elliptical piece is removed, and the incisions gradually converging at either end. If the line of deviation is vertical, a triangular wedge-shaped piece is cut out, the apex being upward and extending as high as possible, and the base reaching to near the base of the septum, where it may be joined by a horizontal incision. Both forms of deviation are frequently met in the same case, and then both incisions are to be made. The excised portion should include the protruding angle. The amount of tissue to be removed can be estimated by the eye. A very important point is to avoid cutting the mucous membrane on the side opposite to the incision. The incisions should be made on the convex side of the septum.

As to the second part of the operation, the method of retaining the septum, Watson insists on the necessity of giving sufficient time for the union by fibrous tissue of the cut edges of the cartilage. He finds that three to four weeks should be allowed, and, therefore, all hard plugs and tubes are inappropriate. He uses Roberts' pin, but made with a flat ring-head covered by a piece

Fig. 1.

of rubber tubing. This he uses in a different way to Roberts, inserting it from the concave side of the septum, just behind its anterior edge, passing it diagonally through to the other side, then across the vertical incisions, then back through the septum to the open side till the head rests within the nostril. It can be worn for three or four weeks without discomfort. The bony septal deviations may be crushed straight with forceps; the pieces override to some extent, and, having none of the resilience of cartilage, can be kept straight by a plug of iodoform gauze. This may be used, as the bony fragments unite more rapidly than cartilage, and may be removed in a week or ten days.

If a horizontal deviation exists low down, it may be impossible to bring the lower fragment into line.

Then, instead of cutting out an elliptical piece along the horizontal line, Watson makes a bevelled incision. The edge of the knife is directed upward and toward the opposite side, and carried through the cartilage, but not the mucous membrane of the opposite side. The incision is made just on the crest of the deviation. Any vertical deviation is cut out as before described. The upper portion is then pressed over toward the other side, where it hooks itself on to the lower, and is thus held in place. This also uses up the redundant tissue. The projecting base can then (or after healing) be removed by the saw.

Fig. 2.—Diagram showing, (1) Deviated septum with line of ncision; (2) Septum replaced, with spur standing; (3) Projecting spur removed.

In the discussion following this article, Asch[14] drew attention to the method advocated by him in 1887, which he had himself performed over one hundred times, and in nearly every case with success. His method is founded on the fact that the only way you can cure is by destroying the resiliency of the septum. Two sections are made as nearly at right angles as possible, and the segments which are made are broken up with the finger. He then inserts a hard rubber tube, and the operation is finished. He formerly plugged the nostrils, but the inconvenience arising from it was so great that he substituted the tubes. The operation is done very quickly, and scissors are employed instead of a knife. One can operate without seeing. Push the finger through after the incision is made, and break down the segments before introducing the tube. One may not get a cure in every case, for no operation can do this; but ninety-five out of every hundred cases will show good results. But we restore the respiratory function, and that is what is wanted.

Carl Seiler pointed out that Asch's operation was a revival of the operation originally introduced by Glascow, who, however, used a punch instead of scissors, so as to make a stellate incision into the septum. But Glascow used a pin, and Seiler states that, in order to make the pin a proper splint, it should be introduced as near the nasal bone as possible, where the cartilaginous septum is inserted. It should run down from the other side, through the septum, and into the cleft of the two superior maxillary bones. Then hammer them fast. This, he said, may sound barbarous and unscientific, but it is really the most successful and satisfactory method of producing a splint for fractured septum, without producing necrosis of either the cartilage of the septum or the superior maxillary bone. The only precaution that has to be observed is that the pin should be cut off short, or level with the skin. He generally allows from forty-eight hours to three days to elapse before he cuts it short. He allows it to project beyond the level of the skin in order to compensate for swelling. There are three of his patients who have the pins in their noses still, because the skin healed over the end, and the pins sunk so that he could not get them out without making a disfiguring scar.

Mackenzie claimed the original idea of Asch's operation for James Bolton, of Richmond.

Delie[13] states that so long as deflections or spurs on the septum cause no inconvenience, they should be left alone. They should be removed (1) when they impede the free circulation of air in the nose; (2) when they give rise to troublesome reflexes; (3) when they are the seat of morbid processes (ulcerations, hæmorrhages); or (4) when they constitute a deformity in a nostril. It is worth noting that, apart from neoplasms of the nose or naso-pharynx, the greatest obstacles to free nasal respiration are situated in the anterior third or half of the respiratory portion of the nose, and less frequently in the posterior quarter. Narrowness of the middle portion of the lower part of the nose does not much interfere with respiration, provided the rest of the nasal cavity is of normal capacity.

In the anterior third of the nose there are two principal causes of obstruction, viz., hypertrophy of the inferior turbinate and spurs on the cartilaginous septum; in the posterior quarter the only cause of obstruction is hypertrophy of the posterior end of the inferior turbinated body. The spurs are mostly, if not entirely, cartilaginous. He advocated removal by means of the gouge.

[8] New York Med. Journ., Oct. 10th, 1896. [9] Ibid., Vol. LXI., p. 139. [10] Annales des Mal. de l'Oreille et du Larynx, XVIII., p. 782. [11] Trans. of the Amer. Lar. Assoc., Vol. IX., p. 202. [12] The Breath of Life, p. 16. [13] New York Med. Journ., Oct. 3rd, 1896. [14] Ibid., Oct. 10th, 1896. [15] Belgian Soc. of Otol. and Laryng., Rep. in Journ. of Laryng., Oct., 1896.

## Figures and Tables

**Fig. 1. f1:**
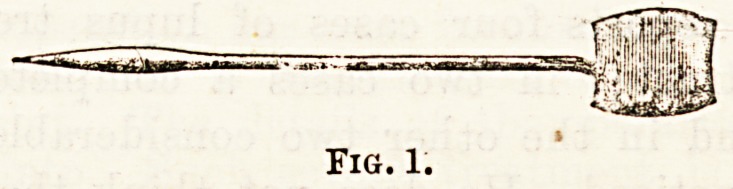


**Fig. 2. f2:**